# HDAC6 Inhibition Reverses Cisplatin-Induced Mechanical Hypersensitivity via Tonic Delta Opioid Receptor Signaling

**DOI:** 10.1523/JNEUROSCI.1182-22.2022

**Published:** 2022-10-19

**Authors:** Jixiang Zhang, Jazzmine M. Junigan, Ronnie Trinh, Annemieke Kavelaars, Cobi J. Heijnen, Peter M. Grace

**Affiliations:** Laboratories of Neuroimmunology, Department of Symptom Research, University of Texas M. D. Anderson Cancer Center, Houston, Texas 77030

**Keywords:** allodynia, CIPN, latent sensitization, opioid receptors

## Abstract

Peripheral neuropathic pain induced by the chemotherapeutic cisplatin can persist for months to years after treatment. Histone deacetylase 6 (HDAC6) inhibitors have therapeutic potential for cisplatin-induced neuropathic pain since they persistently reverse mechanical hypersensitivity and spontaneous pain in rodent models. Here, we investigated the mechanisms underlying reversal of mechanical hypersensitivity in male and female mice by a 2 week treatment with an HDAC6 inhibitor, administered 3 d after the last dose of cisplatin. Mechanical hypersensitivity in animals of both sexes treated with the HDAC6 inhibitor was temporarily reinstated by a single injection of the neutral opioid receptor antagonist 6β-naltrexol or the peripherally restricted opioid receptor antagonist naloxone methiodide. These results suggest that tonic peripheral opioid ligand-receptor signaling mediates reversal of cisplatin-induced mechanical hypersensitivity after treatment with an HDAC6 inhibitor. Pointing to a specific role for δ opioid receptors (DORs), *Oprd1* expression was decreased in DRG neurons following cisplatin administration, but normalized after treatment with an HDAC6 inhibitor. Mechanical hypersensitivity was temporarily reinstated in both sexes by a single injection of the DOR antagonist naltrindole. Consistently, HDAC6 inhibition failed to reverse cisplatin-induced hypersensitivity when DORs were genetically deleted from advillin^+^ neurons. Mechanical hypersensitivity was also temporarily reinstated in both sexes by a single injection of a neutralizing antibody against the DOR ligand met-enkephalin. In conclusion, we reveal that treatment with an HDAC6 inhibitor induces tonic enkephalin-DOR signaling in peripheral sensory neurons to suppress mechanical hypersensitivity.

**SIGNIFICANCE STATEMENT** Over one-fourth of cancer survivors suffer from intractable painful chemotherapy-induced peripheral neuropathy (CIPN), which can last for months to years after treatment ends. HDAC6 inhibition is a novel strategy to reverse CIPN without negatively interfering with tumor growth, but the mechanisms responsible for persistent reversal are not well understood. We built on evidence that the endogenous opioid system contributes to the spontaneous, apparent resolution of pain caused by nerve damage or inflammation, referred to as latent sensitization. We show that blocking the δ opioid receptor or its ligand enkephalin unmasks CIPN in mice treated with an HDAC6 inhibitor (latent sensitization). Our work provides insight into the mechanisms by which treatment with an HDAC6 inhibitor apparently reverses CIPN.

## Introduction

Chemotherapy-induced peripheral neuropathy (CIPN) is a debilitating side effect experienced by 25%-30% of patients receiving treatment for cancer and can persist for months to years (Flatters et al., 2017). Among patients treated with platinum-based agents, the incidence of neuropathy is even higher (70%-100%) (McWhinney et al., 2009; Banach et al., 2017; Zajaczkowska et al., 2019). With limited treatment options available, new approaches to alleviate the symptoms of CIPN are urgently needed (Cavaletti et al., 2019; Colvin, 2019). Histone deacetylase 6 (HDAC6) inhibition has potential as a novel strategy to prevent or reverse peripheral neuropathies induced by cisplatin, paclitaxel, or vincristine (Krukowski et al., 2017; Van Helleputte et al., 2018; Ma et al., 2019; J. Zhang et al., 2022); reversal is especially attractive as it does not interfere with the primary cancer treatment. HDAC6 is predominantly a cytosolic deacetylase that deacetylates nonhistone proteins, including tubulin and heat shock proteins, ultimately regulating processes, such as intracellular protein trafficking and degradation (Hubbert et al., 2002; Bali et al., 2005). Consequently, HDAC6 inhibitors restore axonal mitochondrial content and function after treatment with cisplatin or vincristine (Krukowski et al., 2017; Van Helleputte et al., 2018; Ma et al., 2019; J. Zhang et al., 2022). In contrast to other agents that suppress CIPN in preclinical models (for review, see Flatters et al., 2017), a 2 week course of dosing with an HDAC6 inhibitor persistently reverses peripheral neuropathy induced by cisplatin and paclitaxel (Krukowski et al., 2017; Ma et al., 2019; J. Zhang et al., 2022). We have shown that conditional KO of *Hdac6* in advillin^+^ sensory neurons only partially attenuates mechanical hypersensitivity (Ma et al., 2019), indicating that the pain-resolving effects of HDAC6 inhibitors rely on as yet to be elucidated intercellular communication. In addition, it is not known whether reversal of cisplatin-induced mechanical hypersensitivity by HDAC6 inhibitors represents a true return to baseline or whether hypersensitivity is suppressed by ongoing cell–cell signaling.

There is evidence that the endogenous opioid system contributes to the apparent resolution of pain caused by traumatic nerve injury, inflammation, or a short course of chemotherapy (Corder et al., 2013; Marvizon et al., 2015; Inyang et al., 2021). After pain has spontaneously resolved in these models, inhibition of opioid receptor signaling leads to reinstatement of mechanical hypersensitivity. For example, antagonists for μ, δ, and κ opioid receptors (MOR, DOR, KOR, respectively) reinstate nociceptive hypersensitivity after resolution of inflammatory pain induced by intraplantar complete Freund's adjuvant (CFA) (Corder et al., 2013; Walwyn et al., 2016). The spontaneous resolution of CIPN induced by a short (3 d) course of cisplatin is maintained by coupling between MORs and DORs (heteromers) (Inyang et al., 2021). These findings indicate that, after tissue healing, the resolution of pain depends on transition of the nervous system into a novel state referred to as latent sensitization, in which the suppression of hypersensitivity is maintained by tonic endogenous opioid signaling (Corder et al., 2013; Marvizon et al., 2015; Pereira et al., 2015).

Here, we evaluated whether the persistent reversal of long-lasting mechanical hypersensitivity (>70 d) induced by two 5 d cycles of cisplatin by an HDAC6 inhibitor represents a true return to baseline or a transition to latent sensitization. We show that the reversal of cisplatin-induced mechanical hypersensitivity by an HDAC6 inhibitor is sustained by tonic activation of DOR in peripheral sensory neurons by its endogenous ligand enkephalin.

## Materials and Methods

### Animals

Male and female C57BL/6J mice (8-10 weeks of age) were purchased from The Jackson Laboratory (#000664). Male and female *Hdac6*^−/−^ mice on a C57/Bl6 background were kindly provided by Patrick Matthias (Friedrich Miescher Institute for Biomedical Research, Basel, Switzerland) (Y. Zhang et al., 2008). *Avil*^Cre^::*Oprd1*^fl/fl^ mice were obtained by crossing *Avil*^Cre+/−^ mice (#032536, The Jackson Laboratory) (Zhou et al., 2010) with *Oprd1*^fl/fl^ mice (kindly provided by Gregory Scherrer, UNC School of Medicine) (D. Wang et al., 2018). All mutant mice were genotyped before inclusion in experiments (TransnetYX), and WT littermates were used as controls. Mice were maintained at the University of Texas M. D. Anderson Cancer Center animal facility and group-housed in individually ventilated cages on a regular 12 h light/dark cycle with free access to food and water. All experimental procedures were consistent with the National Institute of Health's *Guide for the care and use of laboratory animals*, and were approved by the M. D. Anderson Animal Care and Use Committee. Mice were randomly assigned to experimental groups, and all analyses were performed by investigators who were blinded to group assignments.

### Drug administration

Cisplatin (#NDC 16729-288-11, TEVA Pharmaceuticals) was diluted in sterile PBS (#21-040-CV, Corning) to a concentration of 0.23 mg/ml and administered intraperitoneally at a dose of 2.3 mg/kg per day for 5 d followed by 5 d of rest and another 5 d of injections (Krukowski et al., 2017). The HDAC6 inhibitor ACY-1083 (Regenacy Pharmaceuticals) was prepared in vehicle consisting of 20% 2-hydroxypropyl-B-cyclodextrin (#297565000, ACROS Organics) and 0.5% hydroxypropyl methylcellulose (#Hy124, Spectrum Chemical) in sterile water (Krukowski et al., 2017). Three days after completion of cisplatin treatment, mice received daily intraperitoneal injections of ACY-1083 at a dose of 10 mg/kg for 2 weeks (Krukowski et al., 2017). Naltrexone hydrochloride (#N3136, Millipore Sigma) was freshly dissolved in sterile PBS and was injected subcutaneously at the nape of the neck at a dose of 3 mg/kg (Marvizon et al., 2015). 6β-Naltrexol hydrate (#N9412, Millipore Sigma) was freshly dissolved in 1% DMSO diluted with sterile H_2_O and was injected subcutaneously at the nape at a dose of 10 mg/kg (Walwyn et al., 2016). Naltrindole hydrochloride (#N115, Millipore Sigma) was freshly dissolved in sterile saline and was injected subcutaneously at the nape with a dose of 3 mg/kg (Walwyn et al., 2016). Naloxone methiodide (#N129, Millipore Sigma) was freshly dissolved in sterile saline and was injected subcutaneously at the nape of the neck at a dose of 5 mg/kg (Z. Wang et al., 2021). Norbinaltorphimine (norBNI; #5080170001, Millipore Sigma) was freshly dissolved in sterile saline and was injected subcutaneously at the nape with a dose of 10 mg/kg (Steinmiller and Young, 2008; Shu et al., 2011). D-Phe-Cys-Tyr-D-Trp-Orn-Thr-Pen-Thr-NH2 (CTOP; #HY-P1329A, MedChemExpress) was freshly dissolved in sterile H_2_O and was injected subcutaneously at the nape with a dose of 3 mg/kg (Gerhold et al., 2015). D-Phe-Cys-Tyr-D-Trp-Arg-Thr-Pen-Thr-NH2 (CTAP; #HY-P1335A, MedChemExpress) was freshly dissolved in sterile H_2_O and was injected subcutaneously at the nape with a dose of 1 mg/kg (Bartlett et al., 2020). Anti-enkephalin antibody (#T-4293, BMA Biomedicals) or control IgG (#I5006, Millipore Sigma) was reconstituted in sterile water at a concentration of 2 μg/μl and intrathecally injected (10 μg per mouse) (Celik et al., 2020).

### Intrathecal injections

Mice were lightly anesthetized with 1.5% isoflurane, and the fur was shaved to expose the skin of the lower back. A 27 G needle was inserted in the interspace between L4 and L5, and a sudden tail flick was used as indication of successful entry of the needle into the intradural space, as we have previously described (Krukowski et al., 2016).

### von Frey test

Mechanical sensitivity was measured using the up and down method with von Frey hairs (0.07, 0.16, 0.4, 0.6, 1.0, and 1.4 g) (Stoelting) as described previously (Chaplan et al., 1994; Krukowski et al., 2017).

### RT-PCR

Mice were killed by CO_2_ (time point for the analysis of changes of *Oprd1*: 24 h after completion of ACY-1083 treatment; time point for validation of *Avil*^Cre^::*Oprd1*^fl/fl^: 8 weeks old after birth) and transcardially perfused with ice-cold PBS. Lumbar DRGs and lumbar spinal cord (L4-L6) were collected and saved in the liquid nitrogen. Total RNA was extracted from these tissues using TRIzol (#15596018, Invitrogen) and converted to cDNA with the high-capacity cDNA reverse transcription kit (#4368813, Applied Biosystems). Gene expression level was measured with the following primers: Mouse-*Penk*-forward: CTA CAG TGC AGG CGG AAT GCA; Mouse-*Penk*-reverse: AGG AGA TCC TTG CAG GTC TCC C; Mouse-*Pomc*-forward: TGG TGC CTG GAG AGC AGC CAG; Mouse-*Pomc*-reverse: GGG GGT TTT CAG TCA GGG GCT G; Mouse-*Pydn*-forward: TCA ACC CCC TGA TTT GCT CC; Mouse-*Pydn*-reverse: TCC AAG AGC TTG GCT AGT GC; Mouse-*Oprd1*-forward: ACC AGA AAG GTG GCT GAG TG; Mouse*-Oprd1*-reverse: TTG ACT GCG ACT GGG GTT AC; Mouse-*Oprm1*-forward: TCT GCC ATT GGT CTG CCC GTA A; Mouse-*Oprm1*-reverse: GAT GAG GAC CGG CAT GAT GAA GGC; Mouse-*Oprk1*-forward: ATG AGT GTG GAC CGC TAC ATT GCT G; Mouse-*Oprk1*-reverse: CAG GAA ACT GCA AGG AGC ATT C, normalized to *Tuba1a*, Mouse-*Tuba1a*-forward: CCA CTA CAC CAT TGG CAA GGA GA; Mouse-*Tuba1a*-reverse: GGA GGT GAA GCC AGA GCC AGT. Quantitative amplification was performed using the SybrGreen (Bio-Rad) with a running program (95°C 3 min and 40 cycles of 95°C for 5 s and 60°C for 30 s).

### RNA ISH (RNAscope)

Mice were killed by CO_2_ (time point for the analysis of changes of *Oprd1*: 24 h after completion of ACY-1083 treatment; time point for validation of *Avil*^Cre^::*Oprd1*^fl/fl^: 8 weeks old after birth) and transcardially perfused with ice-cold PBS. Lumbar DRGs were embedded and freshly frozen in Tissue-Tek OCT Compound (#4583, Sakura). Tissues were cut at 12 μm using a Leica CM3050S cryostat. The RNAscope Fluorescent Multiplex Assay (#320851, Advanced Cell Diagnostics) was used according to the manufacturer's instructions. Briefly, slides were fixed in cold fresh 10% Neutral Buffered Formalin for 15 min and subsequently dehydrated in 50%, 70%, and 100% ethanol. Slides were pretreated with RNAscope Protease IV and hybridized with RNAscope probes following the protocol. Probes used are as follows: Mm-*Oprd1* (DOR, #427371, Advanced Cell Diagnostics), Mm-*Nefh*-C2 (NF200, #443671-C2, Advanced Cell Diagnostics), Mm-*P2rx3*-C3 (P2XR3, #521611-C3, Advanced Cell Diagnostics), and Mm-*Calca*-C2 (CGRP, #578771-C2, Advanced Cell Diagnostics). Sections were imaged using a Nikon A1R Confocal Microscope. The percentages of neurons expressing *Oprd1* (>5 puncta per cell) were calculated by dividing the number of *Nefh*^+^, *P2rx3*^+^, or *Calca2*^+^ neurons positive for *Oprd1* by the total number of respective *Nefh*^+^, *P2rx3*^+^, or *Calca2*^+^ neurons. Individual neurons were defined as ROIs. The mean intensity of each ROI was used to estimate mRNA abundance, and was analyzed using NIS Elements (Nikon).

### Statistics

Minimum group sizes were based on power analysis of results of previous experiments (Krukowski et al., 2017; Ma et al., 2019; J. Zhang et al., 2022). Statistical analysis of the differences between groups was performed in Prism GraphPad9 using one- or two-way ANOVA with Dunnett, Tukey, or Sidak *post hoc* tests as appropriate. Data are expressed as mean ± SEM. *p* < 0.05 was considered statistically significant. All raw data will be made available on request.

## Results

### Endogenous opioid receptor signaling mediates persistent reversal of cisplatin-induced mechanical hypersensitivity after HDAC6 inhibitor treatment

**Figure 1.**
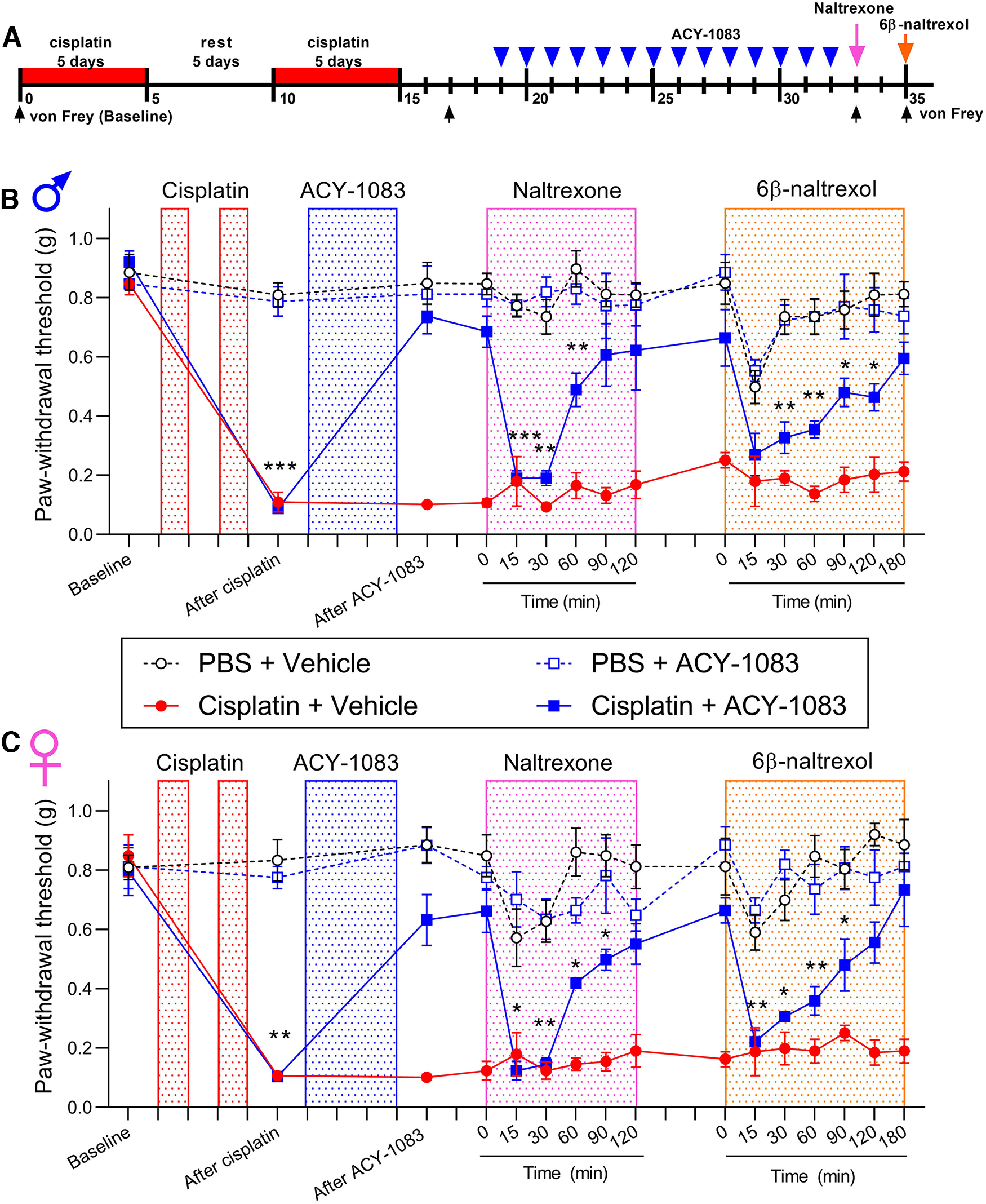
Naltrexone and 6β-naltrexol reinstated cisplatin-induced mechanical hypersensitivity after reversal by HDAC6 inhibitor treatment. ***A***, Timeline of the experimental design and von Frey test. Three days after completion of cisplatin treatment (2.3 mg/kg/d, i.p., 5 d on, 5 d rest, 5 d on), mice were treated with ACY-1083 for 14 consecutive days (10 mg/kg/d, i.p.). Naltrexone (3 mg/kg, s.c.) and 6β-naltrexol (10 mg/kg, s.c.) were injected after completion of ACY-1083. Mechanical hypersensitivity (von Frey testing) was measured at given time points. ***B***, ***C***, Paw withdrawal thresholds from von Frey test in (***B***) male (interaction: *F*_(45,179)_ = 4.915, *p* < 0.0001) and (***C***) female mice (interaction: *F*_(45,180)_ = 4.576, *p* < 0.0001). *p < 0.05; **p < 0.01; ***p < 0.001; cisplatin+ACY-1083 versus PBS (two-way ANOVA with Dunnett *post hoc* test). *n* = 4 mice per group for both males and females.

HDAC6 inhibitors are attractive therapeutics for CIPN because 2 weeks of administration of ACY-1083, a specific HDAC6 inhibitor, results in persistent reversal of cisplatin-induced mechanical hypersensitivity and spontaneous pain (Krukowski et al., 2017; Ma et al., 2019; J. Zhang et al., 2022). In search for a mechanism of action, we first tested whether the reversal of cisplatin-induced hypersensitivity in response to the HDAC6 inhibitor is mediated via sustained activation of opioid signaling. Cisplatin-induced mechanical hypersensitivity was established as described previously (Krukowski et al., 2017): male and female mice received two rounds of cisplatin (2.3 mg/kg/d, i.p., 5 d on, 5 d rest, and 5 d on) (Fig. 1*A*). In line with our previous studies (Krukowski et al., 2017; Ma et al., 2019; J. Zhang et al., 2022), administration of the HDAC6 inhibitor ACY-1083 (10 mg/kg/d, i.p., 14 d) starting 3 d after the last dose of cisplatin resulted in long-lasting reversal of mechanical hypersensitivity (Fig. 1*B*). One day after HDAC6 inhibitor treatment finished, a single injection of naltrexone (3 mg/kg, s.c.) temporarily reinstated mechanical hypersensitivity in male and female mice treated with cisplatin and the HDAC6 inhibitor (Fig. 1*B*,*C*). We did not detect any effect of naltrexone in PBS-treated controls mice or in mice treated with cisplatin only (Fig. 1*B*,*C*). These results suggest that tonic endogenous opioid signaling mediates the reversal effects of the HDAC6 inhibitor on cisplatin-induced hypersensitivity in both sexes.

Naltrexone is an inverse agonist; therefore, the reinstatement of mechanical hypersensitivity could be mediated by either a decrease in the constitutive activity of opioid receptors or by inhibition of the activity of an endogenous agonist. We next used 6β-naltrexol, a neutral opioid receptor antagonist, to test whether endogenous opioids binding to their receptors contribute to the persistent reversal of cisplatin-induced mechanical hypersensitivity in response to administration of the HDAC6 inhibitor. Injection of 6β-naltrexol (10 mg/kg, s.c.) temporarily reinstated mechanical hypersensitivity in male and female mice treated with cisplatin and the HDAC6 inhibitor (Fig. 1*B*,*C*). Neither naltrexone nor 6β-naltrexol altered mechanical thresholds of mice previously treated with cisplatin alone, HDAC6 inhibitor alone, or vehicle controls. These data suggest that binding of endogenous ligand to opioid receptors mediates the persistent reversal of cisplatin-induced mechanical hypersensitivity after HDAC6 inhibitor treatment in both sexes.

### Peripheral opioid signaling mediates the persistent reversal of cisplatin-induced mechanical hypersensitivity after HDAC6 inhibitor treatment

**Figure 2.**
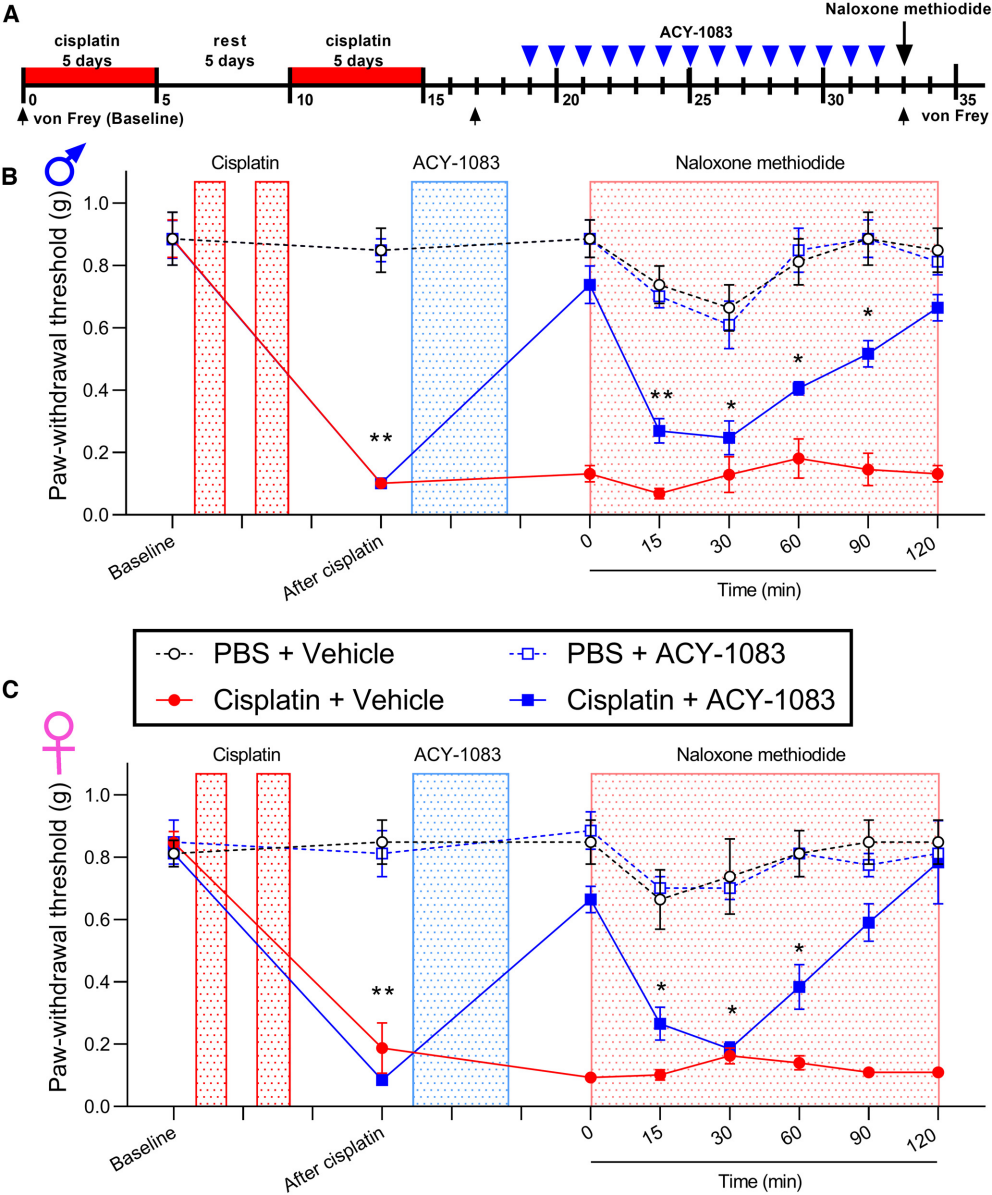
Naloxone methiodide reinstated cisplatin-induced mechanical hypersensitivity after reversal by HDAC6 inhibitor treatment. (***A***) Timeline of the experimental design and von Frey test. Three days after completion of cisplatin treatment (2.3 mg/kg/day, i.p., 5 days on, 5 days rest, 5 days on), mice were treated with ACY-1083 for 14 consecutive days (10 mg/kg/day, i.p.). The peripherally-restricted opioid receptor antagonist naloxone methiodide (5 mg/kg, s.c.) was injected after completion of ACY-1083. Mechanical hypersensitivity (von Frey testing) was monitored at given time points. (***B***-***C***), Paw withdrawal threshold from von Frey test in (***B***) male (interaction: *F*_(21,84)_ = 8.658, *p* < 0.0001) and (***C***) female mice (interaction: *F*_(21,84)_ = 8.287, *p* < 0.0001). *p < 0.05; **p < 0.01; cisplatin+ACY-1083 versus PBS (two-way ANOVA with Dunnett *post hoc* test). *n* = 4 mice per groups for both males and females.

Naltrexone and 6β-naltrexol can act both peripherally and centrally. To evaluate whether peripheral opioid signaling contributes to persistent reversal of cisplatin-induced mechanical hypersensitivity after HDAC6 inhibitor treatment, we tested the peripherally restricted opioid receptor antagonist naloxone methiodide. Mice that received two rounds of cisplatin developed mechanical hypersensitivity, which was reversed by administration of the HDAC6 inhibitor (Fig. 2*A*). One day after HDAC6 inhibitor treatment finished, a single injection of naloxone methiodide (5 mg/kg, s.c.) temporarily reinstated mechanical hypersensitivity in both male and female mice treated with cisplatin and the HDAC6 inhibitor (Fig. 2*B*,*C*). Naloxone methiodide did not alter mechanical thresholds of mice previously treated with cisplatin alone, HDAC6 inhibitor alone, or vehicle controls. These results suggest that sustained activation of opioid receptors in the periphery mediates the reversal effects of the HDAC6 inhibitor on cisplatin-induced hypersensitivity in both sexes.

### Expression of DORs in DRG is decreased by cisplatin and normalized by HDAC6 inhibitor treatment

**Figure 3.**
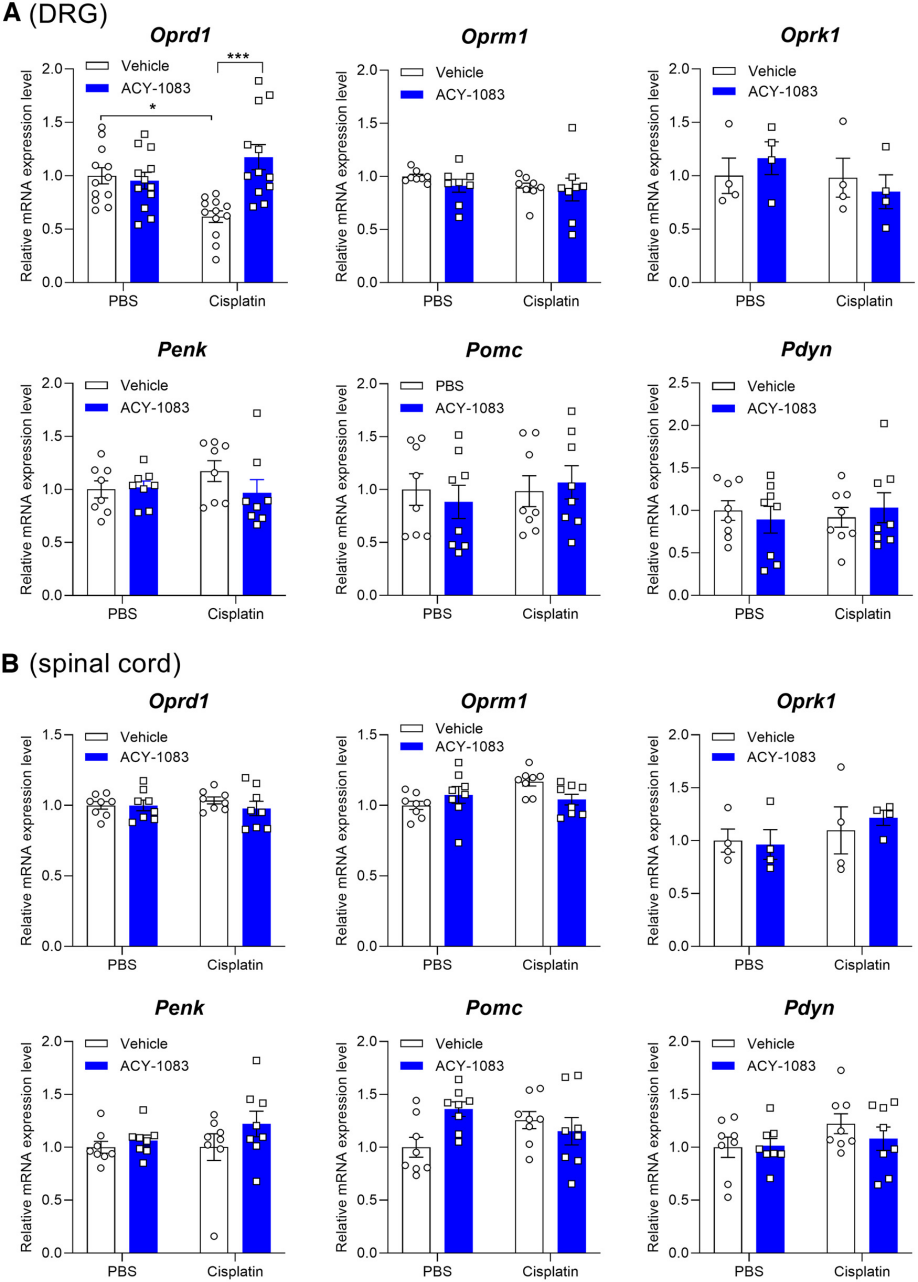
Expression of DORs was decreased by cisplatin and reversed by ACY-1083 in DRG. Relative opioid receptor and opioid peptide precursor gene expression in (***A***) DRG and (***B***) spinal cord. Oprd1: interaction: *F*_(1,44)_ = 12.71, *p* = 0.0009. *p < 0.05; ***p < 0.001; two-way ANOVA with Tukey *post hoc* test. *n* = 12 mice (8 males and 4 females) per group.

The antagonists evaluated in previous experiments inhibit all three opioid receptors (D. Wang et al., 2007). To determine whether expression of specific opioid receptors was altered by cisplatin and/or the HDAC6 inhibitor, we used RT-PCR to quantify the expression levels of *Oprd1*, *Oprm1*, and *Oprk1*. We detected changes only in *Oprd1* expression: cisplatin treatment alone decreased *Oprd1* mRNA levels in the DRG, whereas treatment with the HDAC6 inhibitor normalized expression of *Oprd1* in both sexes (Fig. 3*A*). As mechanical hypersensitivity was reinstated by the neutral opioid receptor antagonist (Fig. 1), suggesting a role for endogenous ligands, we also quantified expression of opioid peptide precursors *Penk*, *Pomc*, and *Pdyn* in DRG. However, gene expression was unchanged by cisplatin or the HDAC6 inhibitor (Fig. 3*A*). Aligning with our finding that a peripheral blockade of opioid receptors was sufficient to reinstate mechanical hypersensitivity, gene expression levels of opioid receptors and opioid peptide precursors did not change in spinal cord as a consequence of cisplatin and/or HDAC6 inhibitor treatment (Fig. 3*B*).

**Figure 4.**
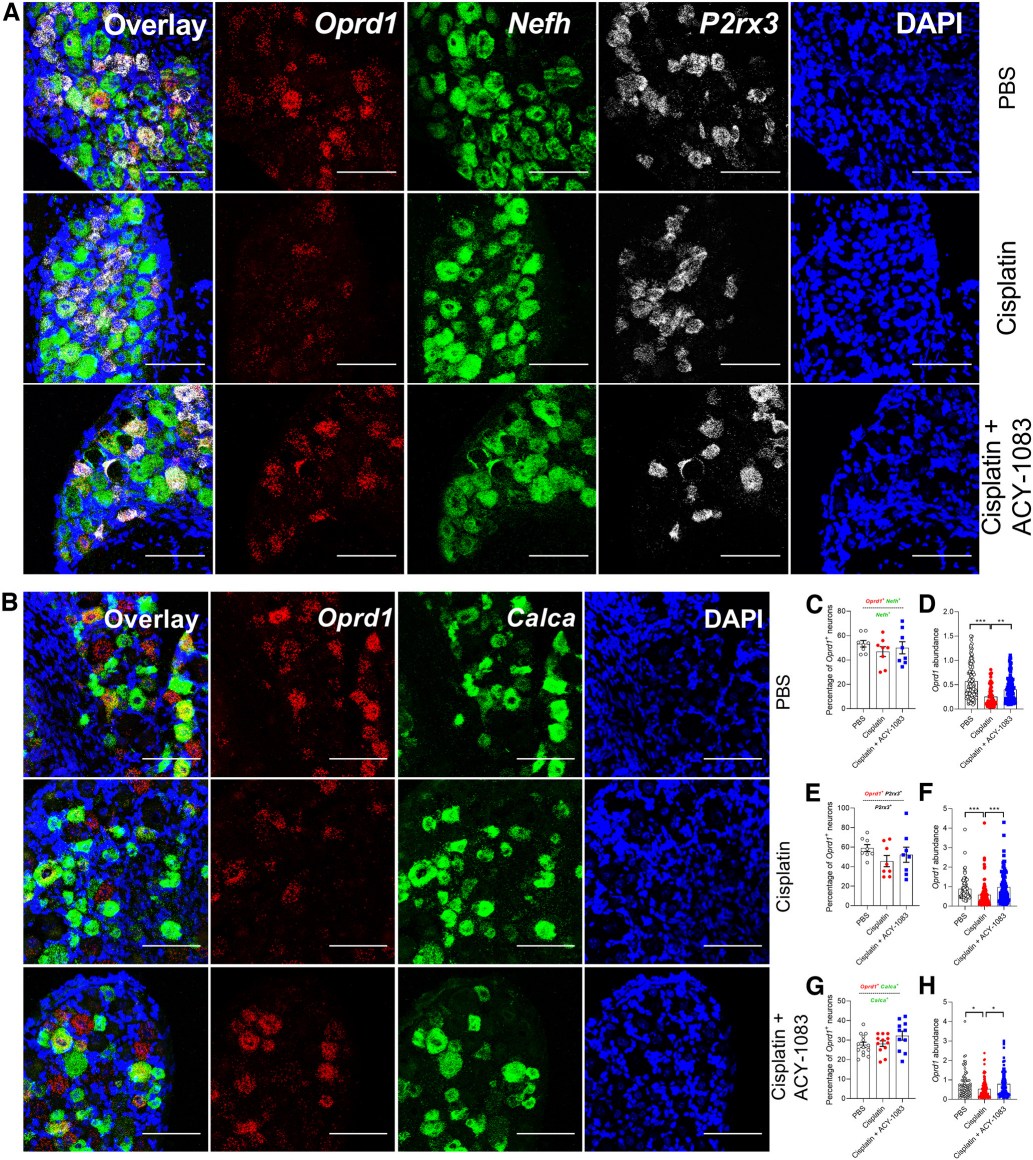
Expression of DORs was decreased by cisplatin and reversed by ACY-1083 in mouse DRG neurons. ***A***, RNAScope was used to detect *Oprd1* mRNA abundance in neurons positive for *Nefh* (encoding NF200 for mechanoreceptors) and *P2rx3*^+^ (encoding P2X3R for nonpeptidergic nociceptors). Scale bar, 100 μm. ***B***, RNAScope was used to detect *Oprd1* mRNA abundance in neurons positive for *Calca2* (encoding CGRP for peptidergic nociceptors). Scale bar, 10 μm. ***C***, Percentages of *Nefh*^+^ DRG neurons also positive for *Oprd1*. *N* = 8 sections from 4 male mice per group. ***D***, *Oprd1* mRNA abundance in *Nefh*^+^ DRG neurons. **p < 0.01; ***p < 0.001; Kruskal–Wallis test with Dunn's *post hoc* test. *n* = 99 neurons for PBS, 67 neurons for cisplatin, and 100 neurons for cisplatin+ACY-1083 from 4 male mice per group. ***E***, Percentages of *P2xr3*^+^ DRG neurons also positive for *Oprd1*. *N* = 8 sections from 4 male mice per group. ***F***, *Oprd1* mRNA abundance in *P2rx3*^+^ DRG neurons. ***p < 0.001 (Kruskal–Wallis test with Dunn's *post hoc* test). *n* = 82 neurons for PBS, 89 neurons for cisplatin, and 96 neurons for cisplatin+ACY-1083 from 4 male mice per group. ***G***, Percentages of *Cgrp*^+^ neurons also positive for *Oprd1*. *N* = 11-14 sections from 4 male mice per group. ***H***, *Oprd1* mRNA abundance in *Cgrp*^+^ DRG neurons. *p < 0.05 (Kruskal–Wallis test with Dunn's *post hoc* test). *n* = 74 neurons for PBS, 102 neurons for cisplatin, and 78 neurons for cisplatin+ACY-1083 from 4 male mice per group.

Next, we used RNAscope analysis to determine whether the cisplatin-induced decrease in *Oprd1* mRNA expression occurred in a specific subset of DRG neurons from male and female mice (Fig. 4*A*,*B*). We found that >50% *Nefh*^+^ neurons (a marker for large-diameter, myelinated neurons) express *Oprd1* (Fig. 4*C*), consistent with a previous report (Bardoni et al., 2014). We did not detect cisplatin-induced changes in the percentage of *Nefh*^+^ neurons expressing *Oprd1*. However, the level of expression of *Oprd1* mRNA in *Nefh*^+^ neurons was significantly decreased by cisplatin, and normalized by treatment with the HDAC6 inhibitor (Fig. 4*C*,*D*). In addition, we observed expression of *Oprd1* by *P2rx3*^+^ neurons (a marker for nonpeptidergic nociceptors) and *Calca*^+^ neurons (a marker for peptidergic neurons) (Fig. 4*A*,*B*), consistent with previous reports (Bardoni et al., 2014). Cisplatin again did not alter the proportion of *P2rx3*^+^ (∼60%) or *Calca*^+^ neurons (nearly 30%) expressing *Oprd1*, but *Oprd1* expression levels were reduced in both subsets (Fig. 4*E–H*). The HDAC6 inhibitor normalized *Oprd1* expression levels (Fig. 4*F*,*H*). Together, these data demonstrate that *Oprd1* expression was decreased by cisplatin in mechanoreceptors, and peptidergic and nonpeptidergic nociceptors, and normalized by HDAC6 inhibitor treatment.

### DOR and enkephalin mediate the persistent reversal of cisplatin-induced mechanical hypersensitivity after HDAC6 inhibitor treatment

**Figure 5.**
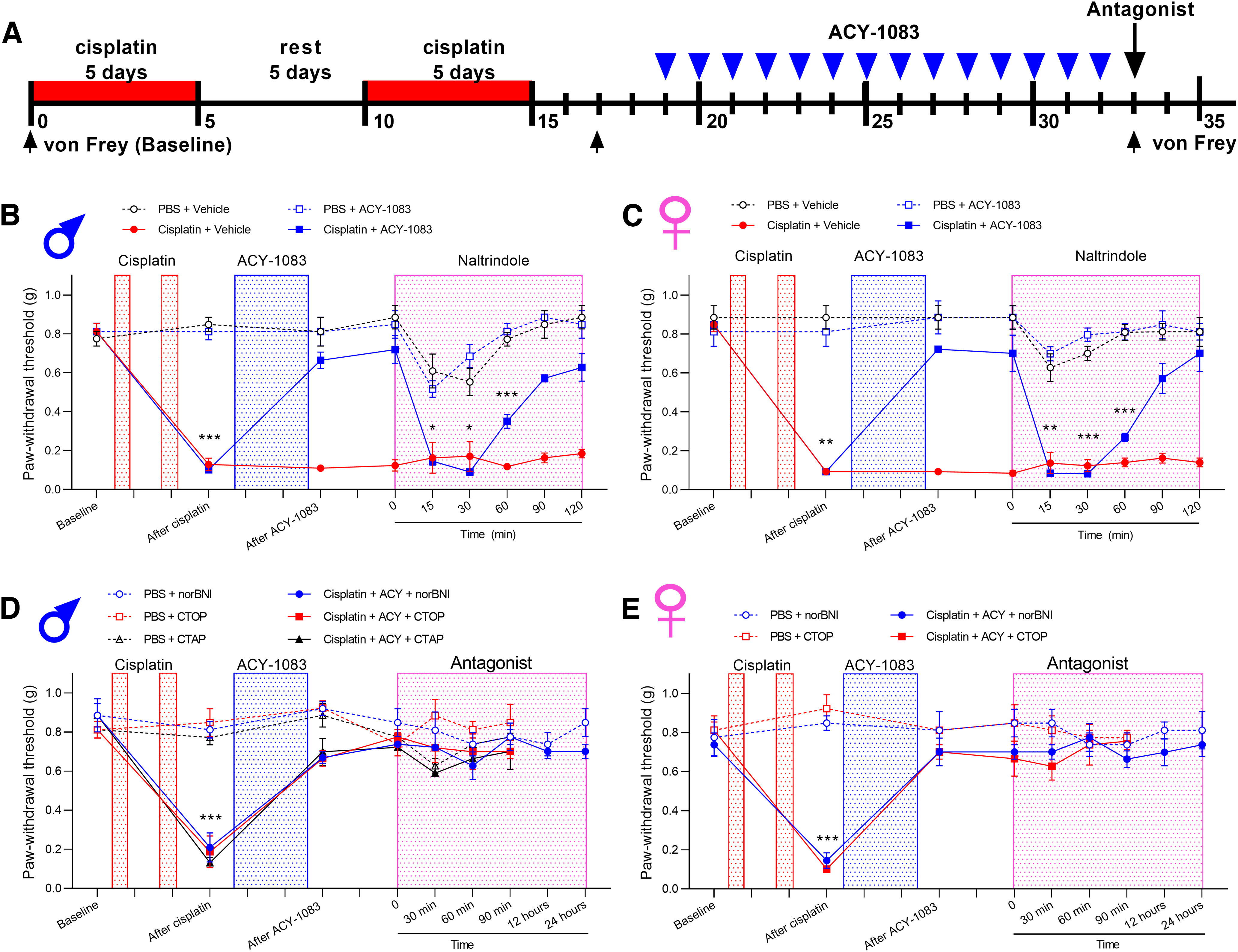
DOR is required for persistent reversal of cisplatin-induced mechanical hypersensitivity by HDAC6 inhibition. ***A***, Timeline of the experimental design and von Frey test. Three days after completion of cisplatin treatment (2.3 mg/kg/d, i.p., 5 d on, 5 d rest, 5 d on), mice were treated with ACY-1083 for 14 consecutive days (10 mg/kg/d, i.p.). The DOR antagonist naltrindole (3 mg/kg, s.c.); MOR antagonist CTOP (3 mg/kg, s.c.) or CTAP (1 mg/kg, s.c.); KOR antagonist norBNI (10 mg/kg, s.c.) was injected after completion of ACY-1083. Mechanical sensitivity (von Frey testing) was monitored at given time points. ***B***, ***C***, Paw withdrawal threshold from von Frey test in (***B***) male (interaction: *F*_(24,96)_ = 9.656, *p* < 0.0001) and (***C***) female mice treated with naltrindole (interaction: *F*_(24,96)_ = 11.99, *p* < 0.0001). *p < 0.05; **p < 0.01; ***p < 0.001; cisplatin+ACY-1083 versus PBS (two-way ANOVA with Dunnett *post hoc* test). *n* = 4 mice per group for both males and females. ***D***, ***E***, Paw withdrawal threshold from von Frey test in (***D***) male and (***E***) female mice treated with MOR and KOR antagonist. ***p < 0.001, cisplatin+ACY-1083 versus PBS (two-way ANOVA with Tukey *post hoc* test). *n* = 4 mice per group for both males and females.

As DOR gene expression in sensory neurons was regulated by cisplatin and ACY-1083, we used the selective DOR antagonist naltrindole to test whether DOR mediates the persistent reversal of cisplatin-induced mechanical hypersensitivity after HDAC6 inhibitor treatment. Mice were treated with cisplatin followed by the HDAC6 inhibitor to normalize mechanical sensitivity (Fig. 5*A*). One day after the HDAC6 inhibitor treatment was finished, a single injection of naltrindole (3 mg/kg, s.c.) temporarily reinstated mechanical hypersensitivity in male and female mice treated with cisplatin followed by the HDAC6 inhibitor (Fig. 5*B*,*C*).

In contrast, mechanical hypersensitivity was not reinstated by single injections of the MOR inverse agonist CTOP or neutral antagonist CTAP, or the KOR inverse agonist norBNI (Fig. 5*D*,*E*). These results collectively suggest that sustained activation of DOR persistently reverses mechanical hypersensitivity in mice of both sexes treated with cisplatin followed by the HDAC6 inhibitor.

**Figure 6.**
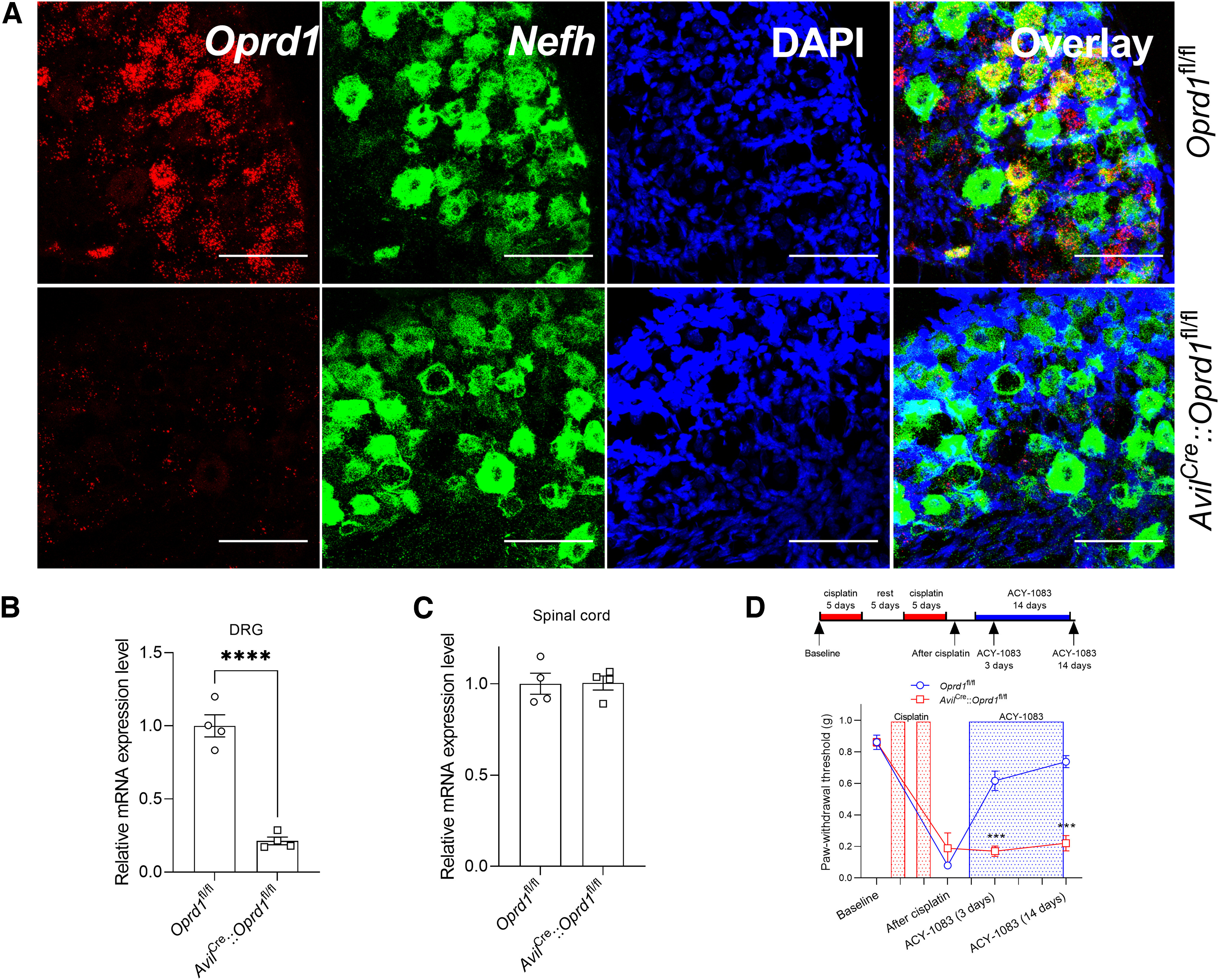
***A***, Validation of *Oprd1* mRNA deletion from DRG neurons using RNAScope in *Avil*^Cre^::*Oprd1*^fl/fl^ mice. Scale bar, 100 μm. ***B***, Validation of *Oprd1* mRNA deletion in DRG (*t*_(6)_ = 9.788), (***C***) but not spinal cords of *Avil*^Cre^::*Oprd1*^fl/fl^ mice using RT-PCR. ****p < 0.0001 (*t* test). *n* = 4 mice (2 males and 2 females) per group. ***D***, Paw withdrawal threshold from von Frey test in *Avil*^Cre^::*Oprd1*^fl/fl^ mice and littermate controls treated with cisplatin and ACY-1083 (interaction: *F*_(3,30)_ = 17.93, *p* < 0.0001). ***p < 0.001 (two-way ANOVA with Sidak *post hoc* test). *n* = 6 mice (3 males and 3 females) per group.

To confirm that DOR in sensory neurons contributes to the reversal of cisplatin-induced mechanical hypersensitivity in response to treatment with the HDAC6 inhibitor, we used *Avil*^Cre^::*Oprd1*^fl/fl^ mice. We verified that *Oprd1* was genetically deleted from sensory neurons, but not the spinal cord (Fig. 6*A–C*). The HDAC6 inhibitor did not reverse cisplatin-induced mechanical hypersensitivity in male or female *Avil*^Cre^::*Oprd1*^fl/fl^ mice, while *Oprd1*^fl/fl^ littermate controls responded similarly to WT mice (Fig. 6*D*). These data indicate that DOR expression in sensory neurons mediates the persistent reversal of cisplatin-induced mechanical hypersensitivity by HDAC6 inhibition.

**Figure 7.**
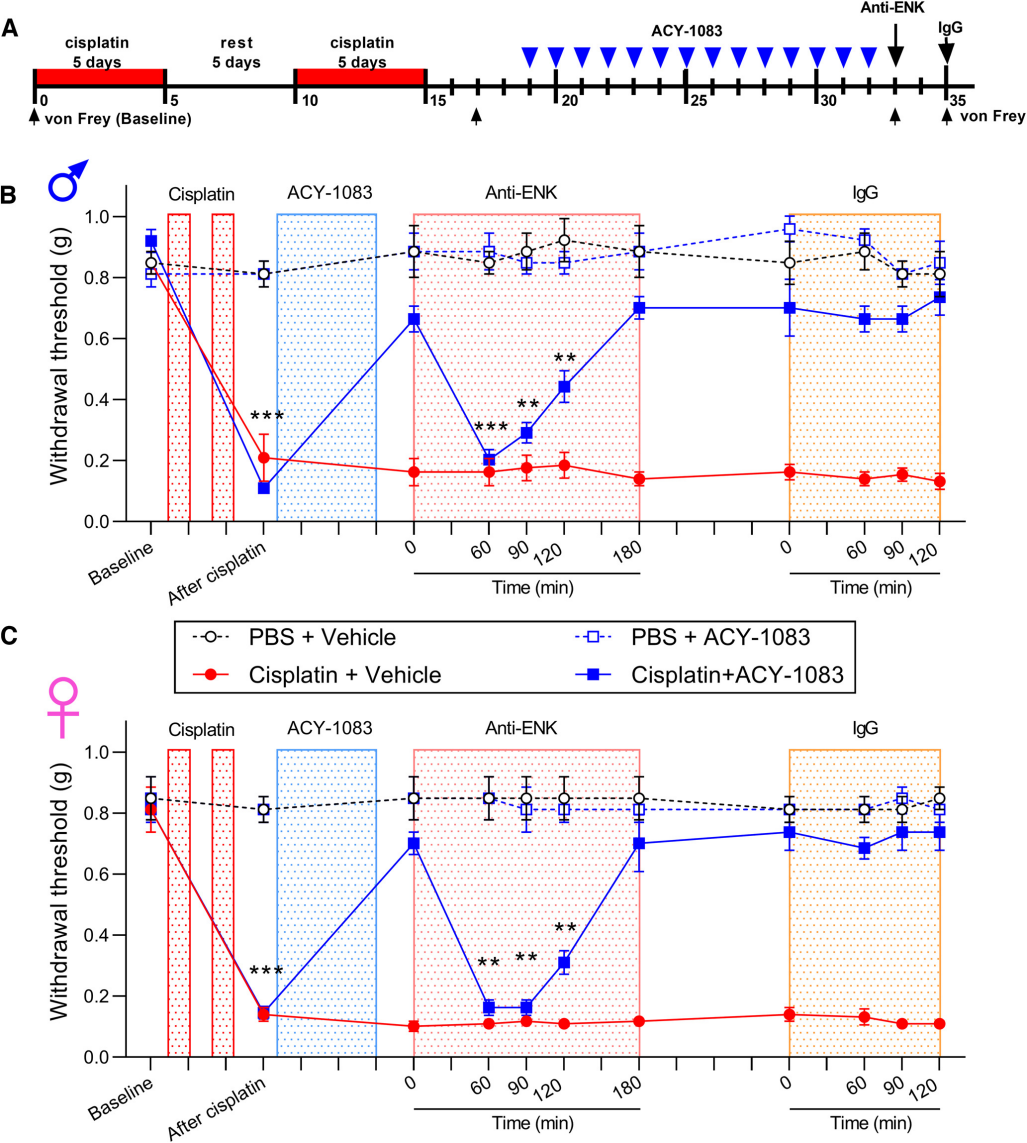
Inhibition of enkephalin reinstated cisplatin-induced mechanical hypersensitivity after reversal by HDAC6 inhibitor treatment. ***A***, Timeline of the experimental design and von Frey test. Three days after completion of cisplatin treatment (2.3 mg/kg/d, i.p., 5 d on, 5 d rest, 5 d on), mice were treated with ACY-1083 for 14 consecutive days (10 mg/kg/d, i.p.). Anti-enkephalin antibody (2 μg) or IgG control was intrathecally injected after completion of ACY-1083. Mechanical hypersensitivity (von Frey testing) was monitored at given time points. ***B***, ***C***, Paw withdrawal threshold from von Frey test in (***B***) male (interaction: *F*_(30,120)_ = 10.39, *p* < 0.0001) and (***C***) female mice (interaction: *F*_(30,120)_ = 13.84, *p* < 0.0001). **p < 0.01; ***p < 0.001; cisplatin+ACY-1083 versus PBS (two-way ANOVA with Dunnett *post hoc* test). *n* = 4 mice per groups for both males and females.

Our finding that the neutral opioid receptor antagonist reinstated mechanical hypersensitivity (Fig. 1) suggested a role for endogenous ligands. We therefore evaluated whether the endogenous DOR ligand enkephalin contributes to persistent reversal of cisplatin-induced mechanical hypersensitivity after treatment with the HDAC6 inhibitor (Fig. 7*A*). Intrathecal injection of neutralizing antibody (2 μg) to inhibit enkephalin in DRG reinstated mechanical hypersensitivity in male and female mice treated with cisplatin and the HDAC6 inhibitor, compared with IgG control (2 μg, i.t.; Fig. 7*B*,*C*). Together, these results suggest that enkephalin-DOR signaling maintains persistent reversal of cisplatin-induced hypersensitivity after HDAC6 inhibition in mice of both sexes.

### Enkephalin-DOR signaling does not mediate prevention of cisplatin-induced mechanical hypersensitivity by HDAC6 inhibition

**Figure 8.**
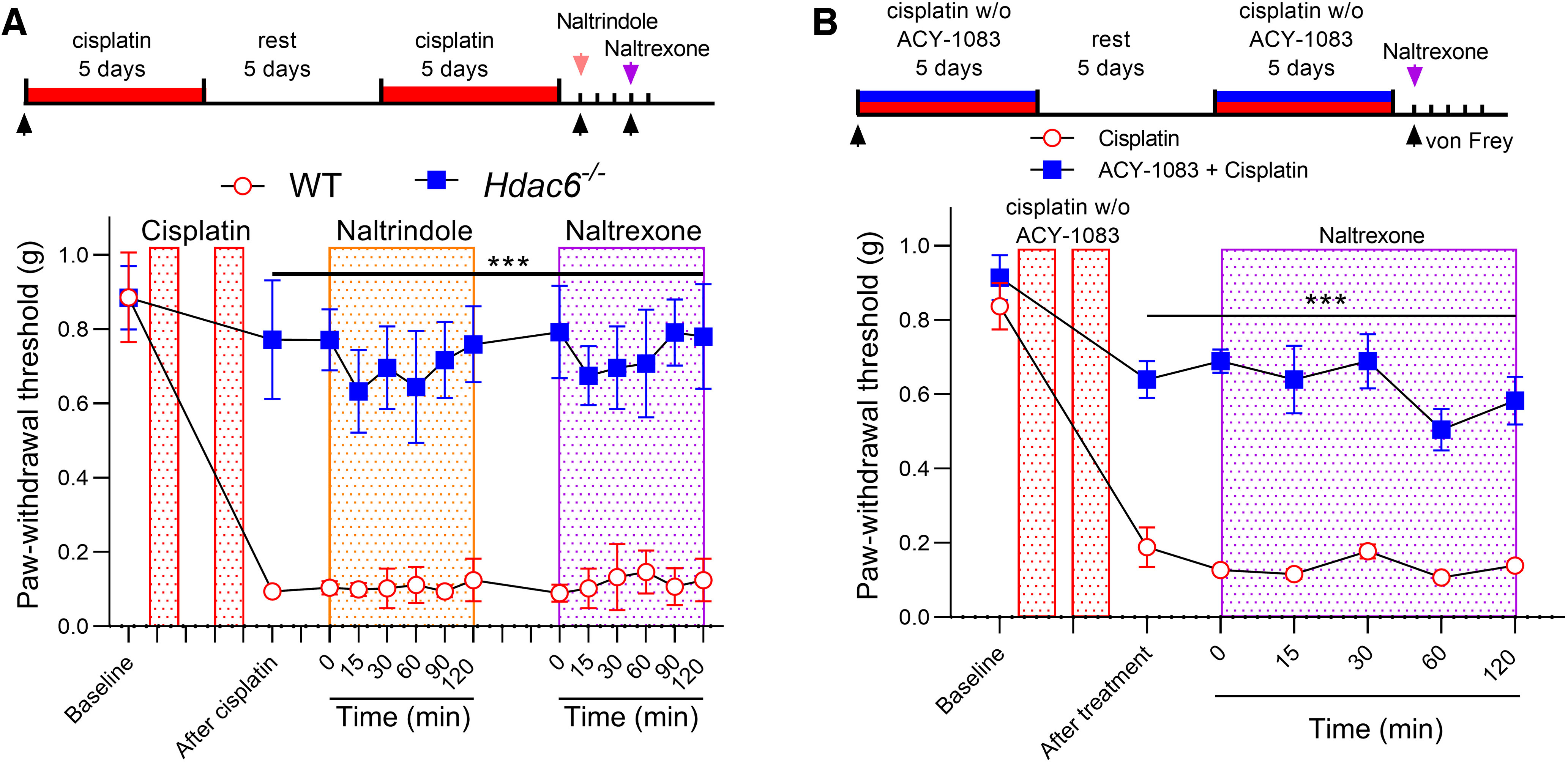
Endogenous opioid signaling did not mediate prevention of cisplatin-induced mechanical hypersensitivity by HDAC6 inhibition. ***A***, One day after completion of cisplatin treatment (2.3 mg/kg/d, i.p., 5 d on, 5 d rest, 5 d on) *Hdac6*^−/−^ and littermate WT mice were treated with naltrindole (3 mg/kg, s.c.) or naltrexone (3 mg/kg, s.c.). Mechanical sensitivity (von Frey testing) was monitored at given time points (interaction: *F*_(13,156)_ = 12.71, *p* < 0.0001). ***p < 0.001 (two-way ANOVA with Sidak *post hoc* test). *N* = 7 mice (4 males and 3 females) for each group. ***B***, One day after coadministration of cisplatin (2.3 mg/kg/d, i.p., 5 d on, 5 d rest, 5 d on) and ACY-1083 (10 mg/kg, i.p., 1 h before each cisplatin injection) was complete, naltrexone (3 mg/kg, s.c.) was injected. Mechanical sensitivity (von Frey testing) was monitored at given time points (interaction: *F*_(6,60)_ = 5.307, *p* = 0.0002). ***p < 0.001 (two-way ANOVA with Sidak *post hoc* test). *n* = 6 male mice for each group.

We previously showed that HDAC6 inhibitors can also prevent cisplatin-induced hypersensitivity, while *Hdac6*^−/−^ mice do not develop mechanical hypersensitivity (Ma et al., 2019). We asked whether tonic endogenous opioid signaling contributes to the prevention of mechanical hypersensitivity after coadministration of the HDAC6 inhibitor with cisplatin. Consistent with our previous findings, cisplatin induced hypersensitivity in male and female WT mice but not in the *Hdac6*^−/−^ mice (Fig. 8*A*) (Ma et al., 2019). However, neither naltrindol nor naltrexone reinstated mechanical hypersensitivity in *Hdac6*^−/−^ mice (Fig. 8*A*). Moreover, naltrexone did not reinstate mechanical hypersensitivity in male mice where cisplatin and the HDAC6 inhibitor were coadministered (Fig. 8*B*). Together, these data indicate that prevention of cisplatin-induced mechanical hypersensitivity by HDAC6 inhibitors is independent of enkephalin-DOR signaling.

## Discussion

**Figure 9.**
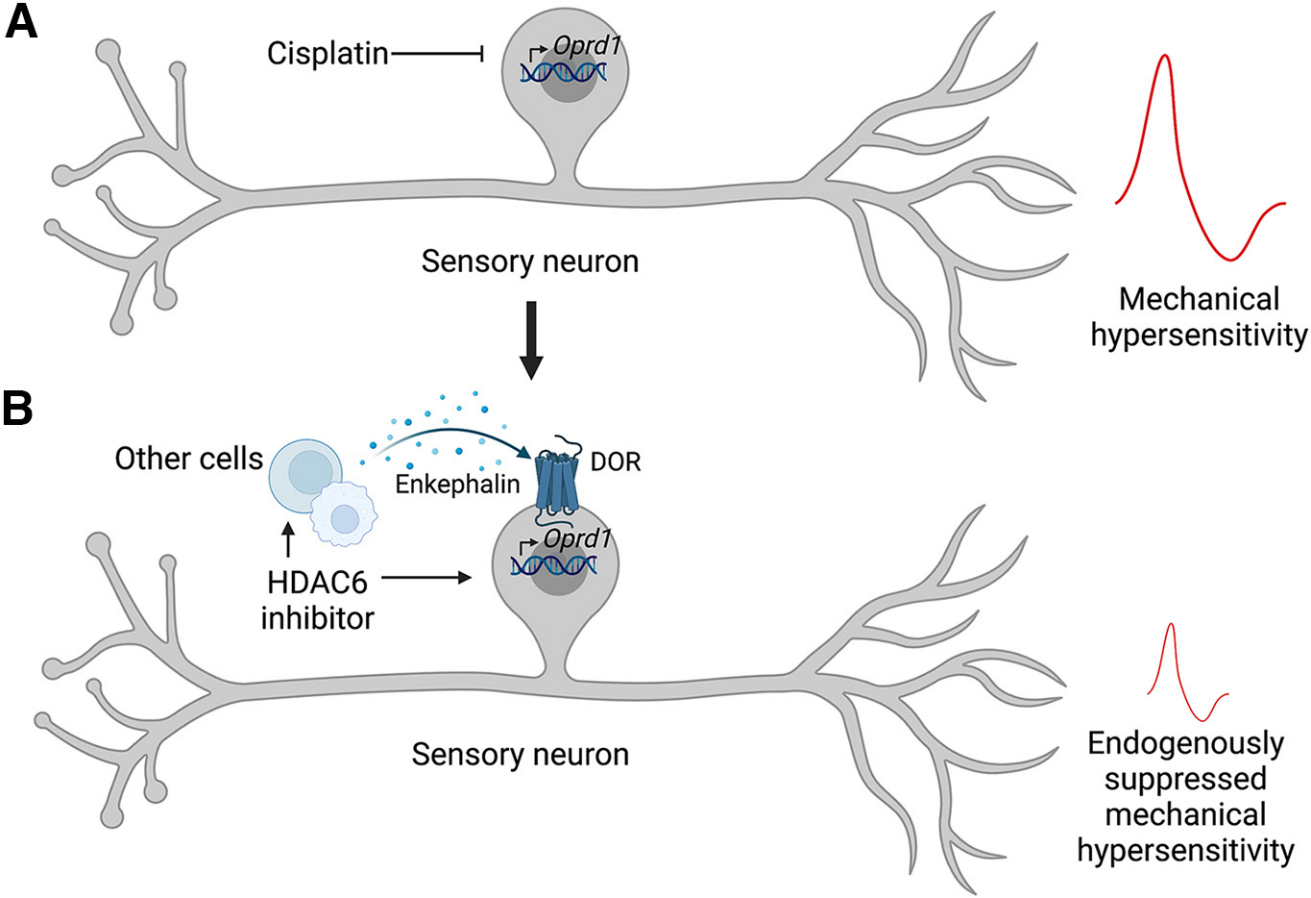
Summary of cellular mechanisms underpinning (***A***) cisplatin-induced hypersensitivity and (***B***) persistent reversal of cisplatin-induced hypersensitivity after treatment with an HDAC6 inhibitor. Created with www.BioRender.com.

We have discovered that DOR expressed by peripheral sensory neurons and its endogenous ligand enkephalin are responsible for apparent resolution of cisplatin-induced mechanical hypersensitivity after treatment with an HDAC6 inhibitor, in both sexes. In this context, the HDAC6 inhibitor therefore generates a state of latent sensitization in which cisplatin-induced mechanical hypersensitivity is tonically suppressed, rather than truly resolved (returned to the pre-injury state), summarized in Figure 9.

Our data show that opioid signaling in the peripheral nervous system tonically suppresses cisplatin-induced mechanical hypersensitivity in response to HDAC6 inhibition. This is different from latent sensitization after spontaneous recovery from inflammatory pain induced by CFA where systemic injection of a peripherally restricted opioid receptor antagonist, naltrexone methobromide, failed to reinstate nociceptive hypersensitivity (Corder et al., 2013). However, in a model of ocular neuropathic pain, spontaneously resolved nociceptive hypersensitivity was reinstated by topical naloxone methiodide, indicating the contribution of peripheral opioid signaling in suppression of ocular pain (Cho et al., 2019). These data suggest that there are injury-specific differences in the opioid responses that suppress pain in states of latent sensitization. These differences may reflect the local needs or microenvironments of the injured tissues, including the local capacity to produce enkephalin and/or express DOR.

We further show that apparent resolution of mechanical allodynia (latent sensitization) following treatment with an HDAC6 inhibitor is dependent on the DOR ligand enkephalin. This again differs from the CFA model, where remission of hypersensitivity was maintained by constitutive MOR activity (Corder et al., 2013) (which had no role in latent sensitization following treatment with an HDAC6 inhibitor). Although the gene encoding proenkephalin is expressed in a subpopulation of human and mouse DRG neurons (Sapio et al., 2020; Tavares-Ferreira et al., 2022), *Penk* mRNA expression was unchanged in DRG of mice treated with cisplatin followed by the HDAC6 inhibitor. These data suggest a source other than sensory neurons, and the cells that secrete enkephalin following treatment with HDAC6 inhibitors will be investigated in future studies. One possible source would be immune cells, which are capable of secreting opioid peptides (Heijnen et al., 1991). For example, repeated interleukin-4 treatment increased the expression of endogenous opioid peptides in macrophages, thereby alleviating nociceptive hypersensitivity because of peripheral nerve injury (Celik et al., 2020; Labuz et al., 2021). In addition, opioid peptides produced by CD4^+^ T cells in the inflamed gut contribute to pain control in models of inflammatory bowel disorder (Boue et al., 2014). It was also reported that opioid peptides produced by T cells suppress pain during late pregnancy (Rosen et al., 2017). The upstream regulators of endogenous opioids in immune cells still need to be defined in the context of treatment with HDAC6 inhibitors. We have previously shown that interleukin-10 also mediates the apparent resolution of cisplatin-induced mechanical hypersensitivity after treatment with an HDAC6 inhibitor (J. Zhang et al., 2022). Interleukin-10 may be upstream of enkephalin production, as this cytokine can regulate secretion of endogenous opioids during neuropathic pain (Wu et al., 2017, 2018). Collectively, these studies reveal that crosstalk between the immune and opioid systems contributes to reduced neuronal hyperexcitability. Such signaling may be exploited to manage chronic pain (Kavelaars and Heijnen, 2021), potentially using HDAC6 inhibitors.

It is yet to be determined how treatment with the HDAC6 inhibitor restores *Oprd1* expression. Although HDAC6 primarily acts in the cytosol (Hubbert et al., 2002; Bali et al., 2005), the normalization of *Oprd1* expression following treatment with the HDAC6 inhibitor points to transcriptional control. *In vitro* studies show that residual nuclear HDAC6 can acetylate histones directly, or indirectly through repression of histone aceytltransferases (Girdwood et al., 2003; Liu et al., 2012; García-Domínguez et al., 2020). HDAC6 may also regulate *Oprd1* indirectly through cytosolic interactions with unknown negative regulators. Future studies will therefore evaluate whether HDAC6 inhibitors increase histone acetylation in the *Oprd1* promoter region. It is also possible that the long duration of treatment with the HDAC6 inhibitor engages other repair mechanisms that indirectly restore *Oprd1* expression (e. g., enhanced interleukin-10 signaling, recovered mitochondrial function).

An intriguing observation is that opioid receptor antagonists did not reinstate mechanical hypersensitivity when the HDAC6 inhibitor was used to prevent neuropathic pain or in *Hdac6*^−/−^ mice. First, this finding suggests that a previous painful experience is necessary for the establishment of latent sensitization. Pain-related behaviors induced by nerve injury, inflammation, or chemotherapy seem to be the trigger for development of latent sensitization, rather than full recovery to baseline; opioid receptor antagonists reinstate hypersensitivity after spontaneous recovery, but not in mice without a previous pain experience (Corder et al., 2013; Marvizon et al., 2015). The duration and/or intensity of the prior painful experience that is necessary to induce a state of latent sensitization after apparent recovery is still an open question. Nonetheless, these data demonstrate that resolution of pain is driven in part by a transition to a novel state that is associated with active opioid signaling and sustained changes in gene expression in the nervous system (latent sensitization). Second, our current results show that tonic endogenous opioid signaling is not required to suppress mechanical hypersensitivity if CIPN was prevented by previous coadministration of the HDAC6 inhibitor with cisplatin. Chronic pain conditions can recur after a period of remission (Stanton et al., 2010), and it has been suggested that apparent remission of pain may reflect a state of latent sensitization, rather than full recovery (Inyang et al., 2021). If latent sensitization increases the risk of CIPN relapse, it may be advantageous to prevent rather than reverse cisplatin-induced mechanical hypersensitivity.

DORs are widely expressed in DRG neurons, as shown in this study and others (Bardoni et al., 2014; Francois and Scherrer, 2018), where they control hyperexcitability mediating nociception. For example, presynaptic DORs suppress glutamate release from mechanoreceptors in the spinal dorsal horn (Bardoni et al., 2014). In addition, inflammatory and neuropathic pain was exacerbated in mice lacking DOR in Na_V_1.8^+^ nociceptors, while acute pain responses remained largely intact (Gaveriaux-Ruff et al., 2011). In this study, we found that cisplatin significantly decreased the expression of *Oprd1* in *Nefh*^+^ neurons. This result suggests that mechanoreceptors may be disinhibited following cisplatin treatment, aligning with the increase in mechanical hypersensitivity. The expression of *Oprd1* in peptidergic (*Calca2*^+^) and nonpeptidergic (*P2rx3*^+^) neurons was also decreased by cisplatin treatment. These results suggest that cisplatin also disinhibits nociceptors. Future studies could evaluate which *Oprd1*^+^ neuronal subtypes mediate hypersensitivity to other pain modalities in CIPN. There is also evidence that *Oprd1* expression is downregulated in DRG in other preclinical models of neuropathic pain (Pokhilko et al., 2020). Reduced *Oprd1* expression could therefore be a contributing factor to low DOR agonist efficacy in clinical pain states (Shiwarski et al., 2017; Spahn and Stein, 2017; Abdallah and Gendron, 2018). Strategies to increase *Oprd1* expression, such as treatment with an HDAC6 inhibitor, may be needed to achieve DOR-mediated analgesia.

In conclusion, we report that 2 weeks of treatment with an HDAC6 inhibitor reverses cisplatin-induced mechanical hypersensitivity by inducing tonic enkephalin-DOR signaling in the peripheral nervous system. HDAC6 inhibition therefore creates a new setpoint to suppress ongoing hypersensitivity, rather than fully reversing neuroplastic changes induced by cisplatin. Further investigation is needed to determine how cisplatin and HDAC6 inhibitors dynamically regulate expression of DORs and enkephalin. Where previous studies have shown that inflammatory and neuropathic pain naturally remit through transition to a state of latent sensitization, we demonstrate that this state can be therapeutically induced to rapidly suppress CIPN long term. Importantly, this approach could be harnessed to manage CIPN and other chronic pain conditions without a need for ongoing treatment.
